# Heart Rate Asymmetry, Its Compensation, and Heart Rate Variability in Healthy Adults during 48-h Holter ECG Recordings

**DOI:** 10.3390/jcm12031219

**Published:** 2023-02-03

**Authors:** Greta Sibrecht, Jarosław Piskorski, Tomasz Krauze, Przemysław Guzik

**Affiliations:** 1 Department of Cardiology–Intensive Therapy, Poznan University of Medical Sciences, Przybyszewskiego 49, 60-355 Poznan, Poland; 2 Institute of Physics, University of Zielona Gora, Szafrana 4a, 65-516 Zielona Gora, Poland

**Keywords:** heart rate variability, heart rate asymmetry, sex differences, 48-h Holter ECG

## Abstract

Heart rate asymmetry (HRA) reflects different contributions of heart rate (HR) decelerations and accelerations to heart rate variability (HRV). In this study, we examined various properties of HRA, including its compensation and HRV, in 48-h electrocardiogram (ECG) recordings in healthy adults. Furthermore, we compared sex differences in parameters used to quantify HRA and HRV. Variance-based and relative HRA and HRV parameters were computed for Holter ECG recordings lasting up to 48 h in 101 healthy volunteers. The median age of the subjects was 39 years, with 47 of them being men. The prevalence of all forms of HRA was statistically different from randomness (*p* < 0.0001). Specifically, HR decelerations contributed >50% (C1d) to short-term HRA in 98.02% of subjects, while HR decelerations contributed <50% to long-term HRA in 89.11% of recordings and to total HRA in 88.12% of recordings. Additionally, decelerations accounted for <50% of all changing heartbeats (Porta’s index) in 74.26% of subjects, and HRA compensation was present in 88.12% of volunteers. Our findings suggest that various HRA features are present in most healthy adults. While men had more pronounced HRA expression, the prevalence of short-, long-term, and total HRA and its compensation was similar in both sexes. For HRV, values of variance-based indices were higher in men than in women, but no differences were found for relative measures. In conclusion, our study references HRA and HRV for longer ECG recordings of up to 48 h, which have become increasingly important in clinical ECG monitoring. The findings can help understand and compare the characteristics of HRA and HRV in patients with different diseases.

## 1. Introduction

Heart rate variability (HRV) is a measure of the fluctuation in the beat-to-beat duration of cardiac cycles, as represented by the RR intervals (the distance between consecutive peaks of R waves in the ECG signal) [1,2]. It reflects heart rate changes (HR) originating from the sinus node.

Numerous physiological mechanisms regulate the depolarization rate of the sinus node. Some examples are direct mechanical effects on the heart, specifically the walls of the right atrium, caused by the breathing [3] or changes in circulating blood triggered by the Bainbridge reflex [4]. Other internal factors influencing sinus node activity include temperature fluctuations [5], electrolyte concentrations (e.g., Na^+^, K^+^, Ca^+2^, Mg^+2^, Cl^−^) [6], hypoxia [7], and various hormones and neuroendocrine substances, such as catecholamines, steroids, including sex steroids, angiotensin, endothelin (endothelin 1, 2, and 3), and cytokines. The balance of sympathetic and parasympathetic activity also contributes [8,9,10]. Additionally, natural external factors, such as the amount and duration of daily light and weather conditions (temperature, winds or rain, tides, moon phases, and solar activity), can also affect sinus node function. Furthermore, changes in HR can also be caused by emotions, tiredness, hunger, or the presence of other people [11,12], as well as environmental pollution and noise [13]. In summary, momentary HR results from many mechanisms and factors, and so does HRV.

RR intervals form cardiac time series. Various mathematical algorithms analyze it to quantify HRV. Poincaré plots (PP) of RR intervals is a variance-based method used for analyzing HRV. It separates total variability into long-term and short-term components based on an identity line separating PP points as HR accelerations or decelerations.

Using PP analysis for HRV, Guzik, and Piskorski have described a physiological phenomenon known as heart rate asymmetry (HRA) [14]. HRA reflects the different contributions of HR accelerations and decelerations to various features of HRV. Specifically, HR decelerations have a larger contribution to the short-term HRV [14] but a smaller contribution to the long-term and total HRV [15]. This difference in contribution to short- and long-term HRV is termed the phenomenon of the HRA compensation [16]. Additionally, Porta et al. [17] have reported that, in general, there are more HR accelerations than decelerations in most people at rest or during the day. Furthermore, Piskorski and Guzik have demonstrated that HR accelerations result in more and longer monotonic runs than decelerations in 24-h ECGs of healthy individuals [18].

The physiological origin of HRA is uncertain. All factors regulating momentary HR may cause its accelerations or decelerations. Some of them are naturally asymmetric [19,20,21,22,23,24]. The delay in the sinus node’s response to vagal tone changes is usually shorter than one second compared to the sympathetic response, which can be over 10–12 s. Inspiration is typically associated with heart rate acceleration, while expiration is associated with heart rate deceleration. Baroreflex sensitivity also differs for blood pressure increases versus reductions. The ascending and descending phases of 10-s pulse pressure oscillations (Meyer waves) are asymmetric. The variability of the atrioventricular conduction is asymmetric [25]. Among other physiological factors contributing to HRA are hormones and cytokines, which can be rapidly released or gradually decline (cortisol, insulin). The influence of regularly repeating and irregular or random factors, such as tides, light exposure, temperature, humidity, noise, and social and emotional activity, is asymmetric too. All of these may alter the rate of sinus node depolarization and differently affect HR decelerations and deceleration.

Regardless of the underlying mechanisms, healthy individuals tend to have more heart rate accelerations than decelerations [17]. These asymmetries can be measured using many variance-, number-, structure-based, and entropy methods and trends.

HRA has been studied in ECG recordings of various lengths, from 1 min to 24 h, in both physiological and clinical studies [14,15,18,26,27,28]. However, it has not been explored in ECGs lasting longer than 24 h. In recent years, longer ECG recordings have become more common in clinical use, but there is no standardization for analyzing HRV or HRA. We decided to use 48-h ECG recordings to investigate the presence of HRA and how it changes and provide possible reference values for HRV and HRA.

Sex differences in HRV are already known. Koenig et al. [29], in their meta-analysis of 172 studies, showed that HRV is better expressed in men than women. However, in this analysis, HRV has been analyzed for time recordings between 15 s and 24 h and never for 48-h ECGs. Studies on HRV provide circumstantial evidence that sex differences may also be present in HRA. So far, a stronger expression of HRA in men, compared to women, has been reported only for 30-min resting ECGs [26]. No other investigation has addressed this issue.

The main objective of this study was to study HRA and its compensation and HRV in healthy individuals in ECGs of 48-h length. Additionally, we studied sex differences in HRA and HRV in such long ECGs in healthy people. Finally, this investigation examined the contribution of short-term HRV to the total HRV (CS) and long-term HRV to the total HRV (CL) and their asymmetric properties.

## 2. Materials and Methods

### 2.1. Participants

We recruited 116 adults between 19 and 60 years old. The inclusion criteria were voluntary participation, signing informed consent, and self-reported good health.

The exclusion criteria were any chronic disease or acute illness in the past three months. Patients were asked about a history of myocardial infarction (MI), cerebral stroke, neoplasm, atrial fibrillation or flutter, pulmonary embolism, heart arrhythmia, and sleep apnea. Individuals with past surgeries or interventions on the cardiovascular, respiratory, or nervous systems were excluded. Regular taking medications, except for hormonal contraception, nutrients, and typical supplements, such as vitamins and minerals, was also an exclusion criterion. Professional athletes were not invited. Abnormal findings in physical examination related to the cardiovascular and respiratory systems also excluded parameters. A technical exclusion criterion was the poor quality of acquired Holter-ECG with a total number of technical artifacts or non-sinus beats exceeding 10% of the Holter recording.

All participants underwent resting 12-lead ECG and transthoracic echocardiography (Vivid9 GE Healthcare, Boston, MA, USA). Fifteen volunteers were excluded for the following reasons: one due to tachycardia over 140 beats/min for more than 10 h of the recording, two due to sleep apnea, one due to newly diagnosed hypertension, one due to Wolff–Parkinson–White syndrome, one due to ischemic heart disease the patient was unaware of, one due to undiagnosed previously dilated cardiomyopathy, and eight due to poor quality of acquired Holter-ECG.

Data from 101 participants were used for further analysis. Holter ECG recordings were stored on a computer using Medilog (Schiller, Switzerland) software and then transferred to an external hard drive. All other data were stored in digital form in the REDCAP software (a project available for scientific research at Poznan University of Medical Sciences at redcap.ump.edu.pl, accessed on 28 December 2022) and later used for mathematical and statistical analyses.

### 2.2. 48-h ECG Holter Recordings

Volunteers underwent a three-channel ECG Holter recording, which lasted up to 48 h and was conducted using the Medilog AR12plus device (Schiller, Switzerland). The recordings were captured at a sampling frequency of 8000 Hz, and the minimum duration of the recording was 36 h. The recordings were first automatically analyzed and then reviewed manually to ensure that all beats were correctly classified. The duration of each RR interval and information about its origin, such as sinus, supraventricular, ventricular, or technical artifact, were exported to MATLAB files for further calculations of HRV and HRA. We utilized publicly available, open-source software (HRAexplorer.com) to calculate the HRA parameters. For the computation of HRV and HRA, we only used RR intervals of sinus origin and duration within the range of 300–1800 ms.

### 2.3. Heart Rate and Heart Rate Variability Measurement

In addition to the mean duration of the RR interval (mean RR), the following HRV parameters were included [2,27,30]:

SDNN—the square root of the total RR intervals variance (standard deviation of normal-to-normal RR intervals as a measure of total HRV);
SD1—the square root of the short-term RR intervals variance, is a measure of short-term HRV that reflects the instantaneous changes between each pair of heartbeats. It is equivalent to the root mean square of the successive differences (rMSSD) between RR intervals but is rescaled by a constant value of the square root of 2. As both measures provide identical physiological and mathematical information, we have chosen to present only SD1 and have omitted rMSSD from this analysis. Any conclusions drawn from SD1 can be interpreted in the same way as if rMSSD had been used;
SD2—the square root of the long-term RR intervals variance (standard deviation measuring the dispersion of points in the Poincare plots of RR intervals along the identity line, a measure of the long-term HRV);
SD2/SD1—the ratio of SD2 to SD1 that measures the balance between the long- and short-term HRV;
CV—the coefficient of variance—the index of total variance normalized to the mean RR multiplied by 100%;
pNN50—the percentage of adjacent normal RR intervals (or normal-to-normal–NN) that differ by more than 50 ms. It is a simple measure of short-term HRV based on counting statistics.

The doubled total variance of RR intervals is the sum of the short-term variance (SD1^2^) and the long-term variance (SD2^2^) [30]:

$$SD1^{2}+SD2^{2}=2\times SDNN^{2}$$

Therefore, we further define CS and CL in the following way:

CS—the contribution of the short-term HRV to the total HRV that is a percentage of the short-term variance (SD1^2^) of RR intervals to doubled total variance of RR intervals (SDNN^2^);
CL—the contribution of the long-term HRV to the total HRV that is a percentage of the long-term variance (SD2^2^) of RR intervals to doubled total variance of RR intervals (SDNN^2^).

The contribution of the short-term variance of RR intervals (CS) to the overall HRV is always inversely proportional to the contribution of the long-term variance of RR intervals (CL). Together, these two contributions sum up to 100% [31]:

CS+CL=100%

CS and CL are like mirrors, and in fact, they convey the same information. Thus, we present only CS to avoid mathematical redundancy without losing any critical physiological information. The interpretation of CS shows how much percentage of the total HRV comes from the short-term variability of RR intervals and how much is the remaining part of CL (i.e., long-term HRV).

### 2.4. Heart Rate Asymmetry Measurement

For HRA analysis, we used methods developed by our group [14,15,16,27]. The following normalized HRA parameters were applied:

For the short-term HRA:

C1d—contribution of HR decelerations to the short-term HRV:

$$C1d\;\left[\%\right]=\frac{SD1d^{2}}{SD1^{2}}\times 100\%$$

and

$$SD1^{2}=SD1d^{2}+SD1a^{2}$$

where:

SD1d^2^—part of SD1^2^ derived from HR decelerations;
SD1a^2^—part of SD1^2^ derived from HR accelerations.

C1a—contribution of HR accelerations to the short-term HRV:

C1d+C1a=100%

Similarly, for the long-term HRA:

C2d—contribution of HR decelerations in long-term HRV:

$$C2d\;\left[\%\right]=\frac{\mathrm{SD}2d^{2}}{\mathrm{SD}2^{2}}\times 100\%$$

and

$$SD2^{2}=SD2d^{2}+SD2a^{2}$$

where:

SD2d^2^—part of SD2^2^ derived from HR decelerations;
SD2a^2^—part of SD2^2^ derived from HR accelerations.

C2a—contribution of HR accelerations to the long-term HRV:

C2d+C2a=100%

Similarly, for total HRA:

CTd—contribution of HR decelerations to total HRV:

$$\mathrm{CTd}\;\left[\%\right]=\frac{{\mathrm{SDNNd}}^{2}}{{\mathrm{SDNN}}^{2}}\times 100\%$$

and

$$SDNN^{2}=SDNNd^{2}+SDNNa^{2}$$

where:

SDNNd^2^—part of SDNN^2^ related to HR decelerations;
SDNNa^2^—part of SDNN-related to HR accelerations.

CTa—contribution of HR accelerations to the total HRV:

CTd+CTa=100%

We used Nd, also known as the Porta index, to analyze the number of HR decelerations and accelerations. Nd measures the proportion of RR decelerations to the total number of changing heartbeats (nd + na):

$$Nd=\frac{nd}{nd+na}$$

where:

nd—the absolute number of HR decelerations;
na—the absolute number of HR accelerations.

We used the following established criteria to compute the rates of various HRA forms [14,15,16,27].

The short-term HRA (HRA1) is present when C1d > 0.5. The long-term HRA (HRA2) exists when C2d < 0.5, and the total HRA (HRAT) is observed when CTd < 0.5. The HRA compensation phenomenon occurs when both the criteria for the presence of short-term and long-term HRA are met, i.e., C1d > 0.5 and C2d < 0.5. Additionally, the HRA of the number of HR decelerations and accelerations is present when Nd < 0.5 (HRAN).

Moreover, for the short- (CS) and long-term (CL) contributions to HRV, we distinguish components derived from HR decelerations and accelerations:

CSa—the contribution of the short-term variance to the total HRV derived from HR accelerations:

$$CSa\left[\%\right]=\frac{SD1a^{2}}{2SDNN^{2}}\times 100\%$$

CSd—the contribution of the short-term variance to the total HRV derived from HR decelerations:

$$CSd\left[\%\right]=\frac{SD1d^{2}}{2SDNN^{2}}\times 100\%$$

and

CS=CSd+CSa

CLa—the contribution of the long-term variance to the total HRV derived from HR accelerations:

$$CLa\left[\%\right]=\frac{SD2a^{2}}{2SDNN^{2}}\times 100\%$$

CLd—the contribution of the long-term variance to the total HRV derived from HR decelerations:

$$CLd\left[\%\right]=\frac{SD2d^{2}}{2SDNN^{2}}\times 100\%$$

and

CL=CLd+CLa

As previously mentioned, CS is a reflection of CL. Similarly, the values of CSd and CSa are also mirrored images of each other. CLd and CLa also add up to 100%, and one can be derived from one other. However, when considering total variability, all four parameters, CSd, CSa, CLd, and CLa, are necessary to calculate one of them. Therefore, all CSd, CSa, CLd, and CLa will be presented.

### 2.5. Statistical Analysis

Due to the non-Gaussian distribution of the data (as determined by the D’Agostino-Pearson test), we used nonparametric Spearman correlation to analyze the associations between various HRV and HRA features and other parameters. Binomial tests were used to study the presence of specific asymmetric features. The data summary is presented as the median and interquartile range (IQR). For comparisons between sexes, we used the nonparametric Mann–Whitney test for continuous data and Fisher’s exact test for dichotomized data. Only *p*-values less than 0.05 were considered statistically significant. Statistical analyses were performed using PQStat Software (PQStat v.1.8.4.138, PQStat Software, Poznan, Poland).

## 3. Results

### 3.1. Clinical Characteristics

The median age of all subjects was 39 years (40 years for women and 39 years for men), and the median body mass index (BMI) was 23.7 kg/m^2^ (22.5 kg/m^2^ for women and 24.5 kg/m^2^ for men). Forty-seven (46.53%) of the participants were men, 16 (15.84%) were current smokers, and 19 (18.81%) were ex-smokers. The median systolic blood pressure for all subjects was 118 mmHg (IQR 114–124), diastolic blood pressure 72 mmHg (IQR 67–79), and HR was 70 beats/min (IQR 65–75).

The median systolic blood pressure for women was 118 mmHg (IQR 114–124), diastolic blood pressure 75 mmHg (IQR 68–80), and HR was 70 beats/min (IQR 65–75). The median systolic blood pressure for men was 117 mmHg (IQR 114–124), diastolic blood pressure was 71 mmHg (IQR 66–78), and HR was 70 beats/min (IQR 63–75). No statistically significant differences between these subgroups were observed.

### 3.2. Descriptors of Global, i.e., 48-h, HRV and HRA

Table 1 presents the results of the 48-h HRV and HRA analysis for all participants. It is worth noting that for the relative measures of HRV, CV is approximately 20%, the average contribution of short-term HRV to total HRV (CS) is less than 1.5%, and for each 1 ms of short-term variability (SD1), long-term variability (SD2) increases by a factor of eight.

### 3.3. Prevalence of Various Forms of HRA and HRA Compensation

Table 2 summarizes the rates of various forms of HRA and compensation in all participants. Short-term HRA (HRA1) was observed in nearly all subjects, and long-term (HRA2) and total HRA (HRAT) were found in most of them. The number of HR decelerations was lower than that of the accelerations (HRAN) in nearly three-fourths of the participants. HRA compensation was present in most individuals. All forms of HRA and its compensation were statistically significant (*p* < 0.0001) and more common than for shuffled data, which were 50% for HRA1, HRA2, HRAT, HRAN, and 25% for HRA compensation.

### 3.4. Comparison of HRV and HRA between Men and Women

Table 3 shows the differences in HRV and HRA between men and women. SDNN, SD2, mean RR, SDNNd, SDNNa, SD1d, SD2d, SD2a, C1d, and CLa were significantly higher in men than in women. Nd, CTd, C2d, and CLd were lower in men than in women, indicating that the expression of HRA was stronger in men. Furthermore, pNN50, CS, CV, SD2/SD1, CSd, and CSa did not differ between the sexes.

### 3.5. Prevalence of Various Forms of HRA and HRA Compensation

Short-term, long-term, and total HRA were present in both men and women. Fisher’s exact test showed no significant differences between the sexes. The details are presented in Table 4.

## 4. Discussion

In this study, we demonstrate that short-term, long-term, total HRA, and HRA compensation are present in the 48-h ECGs of all healthy individuals. Additionally, these phenomena are present in both men and women. Despite the stronger expression of HRA in men, the rates of various HRA forms and compensation are similar for both sexes. Additionally, the contribution of short-term HRV to total HRV (CS) is below 1.5% in the 48-h ECGs. Although many HRV parameters have higher absolute values in men, the relative measures are comparable between the sexes.

### 4.1. HRA in Physiological and Clinical Conditions

HRA quantifies different contributions of HR accelerations and decelerations to HRV. Any internal or external factors and mechanisms influencing the sinus rhythm also affect HRV and HRA. If a physiological challenge or disease alters the function of the sinus node and its controlling mechanisms (e.g., autonomic innervation), HRV and HRA will also change [14,15,17,18,27,28,29].

HRA is a fundamental physiological phenomenon underlying HRV. RR interval prolongations and shortenings generate the heart rate’s variance, structure, trends, and complexity. All these phenomena fall under the HRV umbrella.

Different factors can modify HRA expression. It changes in response to the orthostatic stress [32] and passive lower-limb training [33]. Passive orthostasis increases C1d after active upright positioning compared to supine position [34]. Mina-Paz et al. [35] showed that short-term HRA athletes, compared to non-athletes, had more HR decelerations after the orthostatic challenge. Frank et al. [36] also found that yoga training increased C1d. HRA can also be affected by spontaneous respiratory sinus arrhythmia (beat-to-beat variation in HR in response to breathing). Controlled breathing with different inspiration-expiration ratios of 1:2 vs. 1:1 and 1:2 vs. 2:1 increased C1d [21,37].

Stressful conditions, such as mental stress assessed during open-road driving, reduced HRA [38]. Visnovcova et al. observed that HRA declined in response to stress and increased during relaxation [39]. Kaczmarek et al. demonstrated that positive emotions increased short-term HRA in healthy individuals [40]. Tonhajzerova et al. [41] found that HRA indices were altered in children with attention-deficit hyperactivity disorder—an orthostatic challenge reduced HRA parameters compared to healthy kids.

The mechanisms responsible for HRA are not fully understood. Several physiological conditions and clinical scenarios are complex and multifaceted. They involve direct and indirect autonomic influences, hormones (such as epinephrine and norepinephrine, angiotensin, endothelin, cortisol, aldosterone, triiodothyronine, and insulin) [8,9,10], and other mediators, such as nitric oxide. These factors can impact the activity of the sinus node, the myocardium, and the vascular system and mediate various hemodynamic changes [4]. Additionally, many nutrients (glucose, fatty acids, lactic acid), metabolites (adenosine, ATP, CO_2_), and oxygen influence the heart and circulation and may also contribute to HRA [42]. Variations in potassium and sodium concentrations can also affect HRA’s expression by altering the sinus node’s action potential [6,43].

For all these reasons, the exact mechanisms responsible for HRA (and other HRV indices) are uncertain and cannot be limited to changes in the sympathovagal balance alone. Many HRV parameters are believed to approximate the autonomic modulation of the sinus node activity. For recordings longer than 2–5 min, the original Task Force document [2] stated that HRV should not be used to assess autonomic regulation. The concept that HRV reflects sympathovagal balance or vagal tone has been debated and criticized for over 20 years [44,45]. In an animal model, Marmerstein et al. demonstrated that HRV parameters assumed to reflect vagal tone are not correlated with vagal activity in rats [46]. The same is likely true for HRA, but there is currently no data to confirm this.

HRA has also been studied in clinical settings. Short-term HRA expression was reduced in patients with type 1 diabetes [47] and heart failure [48,49]. Impaired HR microstructure was discovered in patients with obstructive sleep apnea syndrome (OSAS) [50]. Those with more severe forms of OSAS had an increased number of longer deceleration and acceleration runs of lengths five to eight compared to those with mild OSAS. HRA not only changed in response to the effective treatment of severe OSAS and predicted the effectiveness of continuous positive airway pressure therapy in these patients [51]. A reduced number of HR deceleration runs, particularly for two, four, and eight consecutive decelerations, predicted premature death in MI survivors [48]. Reduced deceleration runs were associated with an increased risk of all-cause mortality and cardiovascular and sudden cardiac death in the training group (over 1400 patients) and an independent validation group (over 940 patients). In contrast, post-MI survivors with preserved numbers of deceleration runs of two, four, and eight in a row had the lowest all-cause mortality of <1.8% compared to 24% of those who had a reduced number of deceleration runs of four.

Another study showed that septic newborns [52] had more HR deceleration runs of lengths three and four compared to non-septic newborns. Newborns undergoing stress assessments through heel stimulation and heel stick blood sampling presented with reduced short-term HRA indexes [53]. In patients with the major depressive disorder who also had diabetes, short-term HRA indices were significantly decreased in women [54]. An investigation on patients with gastric cancer revealed that HRA expression was decreased in patients with a higher concentration of biochemical cancer markers [55].

### 4.2. HRA and Its Compensation in Healthy People in ECGs of Different Lengths

So far, neither HRA nor its compensation has been studied in ECG recordings longer than 24 h in healthy individuals. Previously, we have shown that all forms of HRA and its compensation have been present in 24-h ECGs in healthy people [28]. With this study, we demonstrate their presence in the 48-h ECGs.

As in the earlier studies on HRA [14,15,17,26], C1d was over 50%, whereas C2d, CTd, and Nd were below 50%. However, compared to HRA in 5-min, 30-min, and 24-h ECGs, all absolute HRA variance-based parameters for the 48-h ECGs had larger values. In the 24-h recordings [28], the mean values of SD1d, SD2d, and SDNNd were 17.59 ms, 130.53 ms, and 93.25 ms, respectively. In our current study, all these values are higher, i.e., 27 ms for SD1, 224 ms for SD2, and nearly 160 ms for SDNN.

The rates of short- and long-term and total HRA (57%) were significantly different from random effect (rate of 50%) in as short as 1-min ECGs in 241 healthy adults resting in a supine position [15]. In the same study, all three forms of HRA were more common in 10-min ECGs, i.e., 73% for HRA1, 66% for HRA2, and 65% for HRAT. These rates were even higher for 30-min ECGs, i.e., 82.6% for HRA1, 76.4% for HRA2, and 76.4% for HRAT.

Very long ECGs, such as the 24-h or 48-h, are recorded in different conditions rather than in supine rest. Therefore, comparing the 24-h with the 48-h ECGs is more appropriate. Using the 24-h ECGs acquired from 87 healthy adults, we demonstrated that global HRA is a dominant physiological phenomenon as the rate of HRA1 was 83.91%, HRA2 was 80.46%, and HRAT was 79.31% [28]. In the current study, we show that all HRA forms of HRA are even more frequent in the 48-h recordings–HRA1 was present in 98.02% of people, HRA2 in 89.11%, and HRAT in 88.12%. However, a statistically significant increase was noted only for the short-term HRA (83.91% vs. 98.02%, *p* = 0.0006 for binominal proportions). Additionally, using our historical data from [28], we found that compared to the 24-h ECGs, the rate of HRAN was similar (75.86% vs. 74.26, respectively) in the 48-h recordings. In contrast, HRA compensation was significantly (*p* = 0.0108) more frequent (73.56% to 88.12%, respectively) in the 48-h EGCs.

All these studies show that the prevalence of global HRA depends on the length of ECGs and is a widespread phenomenon in most healthy people. From a mathematical and statistical point of view, there is a higher probability of a broader range of HR changes during 48 h than 24 h or a few minutes. From a physiological and clinical point of view, the 48-h ECGs encompass various activities of the studied people, including awakeness, sleep, physical activity, rest, mental challenges, and emotional stress. Unsurprisingly, the absolute values of HRA and HRV parameters have larger values in the 48- than 24-h recordings. It is possible, though, that asymmetric and compensatory mechanisms exist within different time scales. Some of them can be captured only in a longer time, such as 48 h.

### 4.3. HRA and HRV in Men and Women

In 2009, Guzik reported that short-term, long-term, and total HRA expression is stronger in adult men than in women in 30-min recordings [26]. To date, no other study has explored sex differences in HRA.

This study on 48-h ECGs shows that all features of HRA are more strongly expressed in men than in women. SD1d, SD2d, SD2a, SDNNd, SDNNa, C1d, and CLa were higher, while C2d, CTd, Nd, and CLd were lower in men than women. However, the prevalence of all forms of HRA and its compensation are similar between the sexes.

Sex differences in HRV during 24-h recordings have been well-studied and systematically analyzed. Bonnemeier et al. [20] assessed healthy subjects and found that SDNN was significantly lower in women. Umetani et al. [56] showed that HRV is significantly lower in girls and women between the ages of 10 and 29 compared to boys and men of the same age. Similarly, Stein et al. [57] found that, among younger participants, women had significantly lower SDNN. However, this statistical significance disappeared in older subjects. A study of children up to 18 years of age found no differences between boys and girls in SDNN [58]. It suggests that detectable differences in HRV between sexes weaken with age, as confirmed by Voss et al. [59] and Bonnemeier et al. [20].

In 2016, Koenig et al. [29] published a meta-analysis of 172 studies stating that HRV is more strongly expressed in men than women. However, the main limitation of this meta-analysis is that it pooled recordings of different lengths, from 10 cardiac cycles up to 24 h, mixed children with adults, and athletes with non-athletes.

Kashiwagi et al. [60] used 48-h ECG to analyze HRV. Data from their paper were also included in the meta-analysis above. However, these authors only used the first 512 beats of every 30-min segment from the 48-h recordings. In total, they analyzed up to ninety-six of the 512-beat fragments and found no sex differences in the spectral measures of HRV.

Another study by Rodrigues et al., also included in the meta-analysis of Koenig et al., used 39-h recordings [61]. However, these authors only used the first 24 h of the available 39-h recordings. They found higher values of the power of low frequency and SDNN in adolescent boys than girls.

Based on current evidence, sex differences in long-term HRV in 48-h recordings have not been previously explored. Our findings align with previous analyses of shorter recordings. We found that women have a shorter mean RR (and thus higher heart rate) during the 48-h recordings but lower values of variance-based HRV parameters, such as SDNN and SD2. In contrast, relative HRV indices, such as pNN50, SD2/SD1, CV, and CS, are comparable between the sexes.

As measured by CS, the short-term contribution to HRV has not been studied in long-term ECGs. We found that CS in the 48-h recordings is subtle, with a median value that does not exceed 1.5%. In our previous study, CS was around 15% (mean +/− SD 13.4 +/− 6.4%) in ECGs of 30-min duration collected from the healthy people [31]. It suggests that in longer recordings, the effect of the short-term variance of RR intervals on total HRV declines, and most of it is built by long-term effects.

The reasons for these findings are unclear, but it is possible that with more extended time, different influences creating total and long-term HRV suppress the influence of short-term HRV. Similar findings have been observed with spectral HRV analysis. The relative participation of high-frequency power decreases while the power of very low frequency and ultra-low frequency increases in ECGs between 5-min and 24-h ECGs [2]. Phenomena occurring less frequently are clearly visualized with spectral HRV in the lowest frequency ranges. Poincare plots of RR intervals become longer along than wider across the identity diagonal line with the time of the recordings. It means that SD2 is increasing relatively more than SD1 in such cases. As only the short-term (SD1^2^) and long-term (SD2^2^) variances sum up to the total HRV (SDNN^2^), the relative predominance of SD2 over SD1 translates to a declining CS [30].

Other factors may also contribute. The 30-min ECGs from our previous studies were acquired during supine rest, while in the current study, all individuals were monitored for 48 h during normal activities in their everyday environment. They were physically and mentally active, going to work, moving and speaking freely, and could sleep as they pleased. These factors combined are likely responsible for the long-term effects on heart rate and HRV.

### 4.4. Study Limitations

We have studied adults between 19 and 60 years old. All people were carefully examined, including their ECG and echocardiography. As for older people, finding a healthy person over 60 years old may be pretty challenging. Therefore, we cannot extrapolate our conclusions to those older than 60. We studied only healthy individuals, so our results cannot be extrapolated to subjects with clinical conditions. We did not perform the spectral analysis because the interpretation of the 48-h spectral HRV is unknown. Our volunteers come from the Polish population with typical features for the Caucasian race. Thus, any conclusion extrapolated to other ethnic groups should be considered uncertain.

### 4.5. Sampling Frequency

Our previous study with the 24-h ECGs on the monotonic runs revealed that a substantial proportion (between 7–8%) of all consecutive RR intervals have an identical duration [18]. We hypothesized that the so-called neutral runs resulted from the low sampling frequency and that we would observe fewer such runs if the frequency was higher. Thus, we deliberately used a very high sampling frequency of 8000 Hz for this study. This high frequency allows for a practical precision of RR interval identification of 0.125 ms. In comparison, in our previous study with 24-h ECGs, the sampling rate was 200 Hz, and the threshold for measuring RR interval duration was above 5 ms [18].

### 4.6. Novelty, Potential Clinical Meaning, and Conclusions

This study provides several new findings. HRA and HRA compensation are common in healthy people in 48-h ECGs. Although HRA expression is better visible in men, no sex differences in the rate of HRA and HRA compensation are noted. The short-term contribution to total HRV (CS) is minimal and not asymmetric in 48-h ECGs. Only the long-term contributions to total HRV (CLd and CLa) show asymmetry.

The coefficient of variation (CV) of HRV shows the average total variability normalized to the duration of the mean RR interval. Although easy to calculate, it is rarely investigated and never for such long ECG recordings.

Different methods are used to quantify HRV [2,27,30,62,63]. Some of the HRV indices have been documented as risk predictors for various groups of patients.

Among all HRV parameters, SDNN is the simplest and most widely studied prognostic factor in cardiac patients. Kleiger et al. [2] were the first to demonstrate SDNN’s predictive value for mortality in survivors of MI. Subsequent studies have confirmed its prognostic value and often used SDNN as the reference predictor in post-infarction populations [48,64].

In 1992, Bigger et al. [65] showed that spectral HRV analysis (total power and powers in the ultra-low, very low, low, and high-frequency ranges) identified MI survivors at higher risk for all-cause mortality, cardiac mortality, and arrhythmic mortality. Farrel et al. [66] reported the prognostic value of the triangular interpolation of normal-to-normal RR intervals for life-threatening arrhythmia or sudden death in survivors of MI.

In 1999, Schmidt et al. described a new phenomenon of heart rate turbulence (HRT) triggered by premature ventricular beats. The reduced expression of HRT quantified by a combination of abnormal turbulence onset and turbulence slope in 24-h ECGs was associated with increased all-cause mortality in survivors of MI [67]. Next, in 2006, Bauer et al. [68] developed a novel method–phase rectified signal averaging. Using 24-h Holter ECGs from patients after MI, they demonstrated that deceleration and acceleration capacities predicted premature all-cause death in three MI survivors from the Munich, London, and Oulu groups. Deceleration capacity is considered the strongest single HRV index for predictive purposes. In cooperation with Georg Schmidt from Munich and Marek Malik from London, our group has shown HR deceleration runs, and acceleration runs (both describing the HRA microstructure) also predicted different modes of death in patients after acute MI [48].

For patients with congestive heart failure, the data is more limited. However, in the UK Heart Study, Nolan et al. showed that a reduced SDNN predicts all-cause mortality and death related to heart failure [69].

Ponikowski et al. studied several HRV parameters as potential predictors in patients with congestive heart failure. They found that reduced SDNN, the standard deviation of five-minute RR intervals (SDANN), and the power of low frequency were independent predictors of mortality. Importantly, their predictive value was independent of NYHA functional class, left ventricular ejection fraction (LVEF), peak oxygen consumption, and ventricular tachycardia on 24-h Holter recordings [70]. In the MUSIC (Muerte Subita en Insuficiencia Cardiaca) study, Cygankiewicz et al. demonstrated that both SDNN and turbulence slope, in particular, predict mortality in patients with LVEF > 35% [71,72]. When combined with other clinical information, Voss et al. showed that indices from nonlinear dynamics might improve risk prediction in such patients [73]. The low-to-high frequency power ratio was identified as an independent risk predictor for the mortality [74].

Unfortunately, long, 48-h ECGs have not been used for prediction purposes in post-infarction or heart failure patients, and the predictive value of any HRV or HRA computed from such long ECGs is unknown.

Our findings may help define reference values for the presented HRV and HRA parameters for 48-h ECGs. They could be useful in determining normal ranges for clinical investigations on various groups of patients at increased risk of death or studying different drug therapies or non-pharmacological interventions.

Nowadays, longer ECG recordings are more attainable than in the past. For 48-h Holter ECGs, this study is the first to summarize HRV, confirm the existence of HRA and HRA compensation, and investigate sex differences in these phenomena.

It should be noted that longer recordings lasting up to several weeks have become available. The results of this study cannot be extrapolated to such recordings, and new studies should be conducted to explore HRV and HRA for longer than 48 h of ECGs. 

## Figures and Tables

**Table 1:** HRV and HRA parameters for all studied subjects (n = 101) from 48-h ECG recordings of carefully selected healthy individuals.

Parameter	Median	IQR
SDNN [ms]	159.83	140.57–184.31
SD1 [ms]	27.29	21.00–35.41
SD2 [ms]	224.83	197.48–257.68
pNN50 [%]	12.26	6.09-21.42
Mean RR interval [ms]	809.83	762.69–865.10
SDNNd [ms]	110.47	98.43–126.79
SDNNa [ms]	115.18	100.16–134.83
SD1d [ms]	21.11	15.48–27.32
SD1a [ms]	17.92	13.42–23.28
SD2d [ms]	155.55	138.13–177.98
SD2a [ms]	162.21	140.50–188.62
Nd [%]	48.93	47.97–50.00
CS [%]	1.43	1.03–2.20
SD2/SD1	8.31	6.67–9.78
CV [%]	19.80	17.54–22.78
CTd [%]	48.05	46.94–49.30
C1d [%]	57.31	54.26–59.84
C2d [%]	47.95	46.73–49.27
CSd [%]	0.85	0.95–1.25
CSa [%]	0.64	0.45–0.96
CLd [%]	47.25	45.72–48.56
CLa [%]	51.27	50.14–52.15

Abbreviations: C1d—the contribution of HR decelerations to the short-term HRV; C2d—the contribution of HR decelerations in long-term HRV; CLa—the contribution of the long-term variance to the total HRV derived from HR accelerations; CLd—the contribution of the long-term variance to the total HRV derived from HR decelerations; CS—the contribution of the short-term variance to the total HRV; CSa—the contribution of the short-term variance to the total HRV derived from HR accelerations; CSd—the contribution of the short-term variance to the total HRV derived from HR decelerations; CTd—the contribution of HR decelerations in total HRV; CV—the coefficient of variation of RR intervals; IQR—interquartile range; Nd—the contribution of HR decelerations in number of all changing RR intervals; pNN50—percentage of successive RR intervals that differ by more than 50 ms; RR—a distance between consecutive peaks of R waves in the ECG signal; SD1—the square root of the short-term RR intervals variance; SD1a—the square root of the short-term RR intervals variance derived from accelerations; SD1d—the square root of the short-term RR intervals variance derived from decelerations; SD2—the square root of the long-term RR intervals variance; SD2/SD1—the ratio of SD1 to SD2; SD2a—the square root of the long-term RR intervals variance derived from accelerations; SD2d—the square root of the long-term RR intervals variance derived from decelerations; SDNN—the square root of the total RR intervals variance; SDNNa—the square root of the total RR intervals variance derived from accelerations; SDNNd—the square root of the total RR intervals variance derived from decelerations.

**Table 2:** The prevalence of short-term, long-term, total HRA, and HRA compensation in the study group (n = 101) of carefully selected healthy individuals during 48-h ECG recordings.

	N	%	*p*-Value
HRA1	99	98.02%	<0.0001
HRA2	90	89.11%	<0.0001
HRAT	89	88.12%	<0.0001
HRAN	75	74.26%	<0.0001
HRAcomp	89	88.12%	<0.0001

Abbreviations: HRA1—short-term HRA; HRA2—long-term HRA; HRAT—total HRA; HRAN—HRA for the condition in which the number of HR decelerations < 50% of all changing RR intervals of sinus origin.

**Table 3:** HRV and HRA parameters for all studied men (n = 47) and women (n = 54) from 48-h ECG recordings of carefully selected healthy individuals.

Parameter	Men		Women		*p*-Value (M-W)
	Median	IQR	Median	IQR	
SDNN [ms]	173.57	153.24–190.49	152.72	130.86–180.89	0.0220
SD1 [ms]	30.61	23.29–38.31	25.00	18.94–33.87	0.0570
SD2 [ms]	243.73	214.19–267.06	215.14	184.04–253.79	0.0204
pNN50 [%]	13.27	7.48–23.05	10.77	5.04-20.18	0.2595
Mean RR interval [ms]	849.14	780.62–910.48	784.23	744.51–827.61	0.0003
SDNNd [ms]	118.18	104.83–131.48	106.60	91.86–124.85	0.0267
SDNNa [ms]	124.81	110.43–137.83	110.19	93.17–130.63	0.0190
SD1d [ms]	23.28	17.38–29.44	17.83	14.00–25.91	0.0240
SD1a [ms]	18.27	14.69–24.09	16.41	12.56–22.29	0.1659
SD2d [ms]	165.93	146.52–184.07	149.54	129.15–172.09	0.0240
SD2a [ms]	175.86	155.10–193.48	155.18	131.02–183.97	0.0194
Nd [%]	48.47	47.46–49.70	49.35	48.42–50.38	0.0187
CS [%]	1.67	1.06–2.34	1.34	1.03–2.09	0.3929
SD2/SD1	7.66	6.46–9.68	8.57	6.85–9.80	0.3891
CV [%]	19.98	18.28–23.20	19.68	17.28–22.29	0.5605
CTd [%]	47.52	46.81–48.75	48.54	47.58–49.39	0.0312
C1d [%]	58.71	56.40–61.49	55.68	52.86–58.72	0.0008
C2d [%]	47.28	46.48–48.71	48.47	47.43–49.37	0.0286
CSd [%]	0.98	0.60–1.41	0.73	0.59–1.12	0.1877
CSa [%]	0.65	0.43–0.92	0.59	0.48–0.95	0.8090
CLd [%]	46.50	45.33–48.38	47.92	46.36–48.72	0.0442
CLa [%]	51.68	50.84–52.35	50.85	49.89–51.93	0.0258

Abbreviations: C1d—the contribution of HR decelerations to the short-term HRV; C2d—the contribution of HR decelerations in long-term HRV; CLa—the contribution of the long-term variance to the total HRV derived from HR accelerations; CLd—the contribution of the long-term variance to the total HRV derived from HR decelerations; CS—the contribution of the short-term variance to the total HRV; CSa—the contribution of the short-term variance to the total HRV derived from HR accelerations; CSd—the contribution of the short-term variance to the total HRV derived from HR decelerations; CTd—the contribution of HR decelerations in total HRV; CV—the index of total variance normalized to the mean RR x100%; IQR—interquartile range; Nd—the contribution of HR decelerations in number of all changing RR intervals; pNN50—percentage of successive RR intervals that differ by more than 50 ms; RR—a distance between consecutive peaks of R waves in the ECG signal; SD1—the square root of the short-term RR intervals variance; SD1a—the square root of the short-term RR intervals variance derived from accelerations; SD1d—the square root of the short-term RR intervals variance derived from decelerations; SD2—the square root of the long-term RR intervals variance; SD2/SD1—the ratio of SD1 to SD2; SD2a—the square root of the long-term RR intervals variance derived from accelerations; SD2d—the square root of the long-term RR intervals variance derived from decelerations; SDNN—the square root of the total RR intervals variance; SDNNa—the square root of the total RR intervals variance derived from accelerations; SDNNd—the square root of the total RR intervals variance derived from decelerations.

**Table 4:** The prevalence of short-term, long-term, total HRA, and HRA compensation in all studied men (n = 47) and women (n = 54) from 48-h ECG recordings of carefully selected healthy individuals.

	Women			Men			*p*-Value for Sex Comparisons *
	N	%	p	N	%	p	
HRA1	53	98.15%	<0.0001	46	97.87%	<0.0001	1
HRA2	47	87.04%	<0.0001	43	91.49%	<0.0001	0.5371
HRAT	46	85.19%	<0.0001	43	91.49%	<0.0001	0.3726
HRAN	36	66.67%	0.0143	39	82.98%	<0.0001	0.0714
HRAcomp	46	85.19%	<0.0001	43	91.49%	<0.0001	0.3726

* Fisher exact test. Abbreviations: HRA1—short-term HRA; HRA2—long-term HRA; HRAcomp—HRA compensation; HRAT—total HRA; HRAN—HRA for the condition in which the number of HR decelerations is below 50% of all changing RR intervals of sinus origin.

## Data Availability

The data presented in this study are available on request from the corresponding author. The data are not publicly available due to the sensitive nature of the data.
